# Endolymphatic hydrops and its association with Magnetic Resonance Imaging and serum vitamin D levels

**DOI:** 10.1016/j.bjorl.2026.101858

**Published:** 2026-06-16

**Authors:** Tao Lin, Xiaoting Wang, Luguang Zhang, Jing Li, Ling Ding

**Affiliations:** a Shandong Second Provincial General Hospital, Department of Neurosurgery, Jinan, Shandong, China; b Shandong Provincial Hospital Affiliated to Shandong First Medical University, Jinan, China & The Second Affiliated Hospital of Shandong First Medical University, Department of Otorhinolaryngology-Head and Neck Surgery, Tai’an, China; c Shandong Provincial Hospital Affiliated to Shandong First Medical University, Department of Medical Imaging, Jinan, China; d Shandong Provincial Hospital Affiliated to Shandong First Medical University, Department of Otorhinolaryngology-Head and Neck Surgery, Jinan, China

**Keywords:** Endolymphatic hydrops, Vitamin D, Gadolinium-enhanced delayed magnetic resonance imaging

## Abstract

**Objective(s):**

Endolymphatic hydrops is associated with factors such as autoimmune responses and vascular issues. Vitamin D plays a vital role in immune regulation and is associated with various inflammatory conditions. However, evidence regarding the correlation between endolymphatic hydrops and vitamin D is lacking. We clarified the correlation between vitamin D and endolymphatic hydrops to explain clinical symptoms and intervene in endolymphatic hydrops formation and progression.

**Methods:**

Intravenous gadolinium-enhanced delayed magnetic resonance imaging three-dimensional-fluid-attenuated inversion recovery technique was used to screen unilateral endolymphatic hydrops patients. Vestibular and cochlear hydrops were categorized as follows: 0 = normal, 1 = mild hydrops, and 2 = severe hydrops. Values of low, middle, and high-frequency bands of pure tone audiometry (low-frequency-, middle-frequency-, and high-frequency-pure tone audiometry); serum 25(OH)D3 level; slow phase velocity of bilateral ears in the caloric test; and summating potential to action potential ratio of affected ears in electrocochleography were documented. Correlations between vestibular-cochlear hydrops grade and these examinations were further analyzed.

**Results:**

Overall, 82 endolymphatic hydrops patients were included. The grade of vestibular-cochlear hydrops was significantly correlated with the summating potential to action potential ratio and slow phase velocity of the affected ear. A significant linear correlation was observed between the vitamin D level and contralateral ear slow phase velocity and low-frequency-pure tone audiometry value (*r* = 0.236 and -0.323, respectively). High-frequency-pure tone audiometry and vitamin D levels exhibited correlations with cochlear hydrops (*U* = 15.06, p = 0.001) and vestibular hydrops (*F* = 4.394, p = 0.016), respectively.

**Conclusions:**

Vitamin D is directly associated with contralateral slow phase velocity and low-frequency-pure tone audiometry, suggesting its correlation with episodic vertigo and fluctuating hearing loss. Early vitamin D supplementation may improve fluctuating hearing loss and vertigo in Meniere’s disease patients with endolymphatic hydrops.

**Level of evidence:**

4.

## Highlights

• Endolymphatic hydrops is most prevalent in Meniere’s disease.
• VD is directly associated with contralateral SPV and LF-PTA.
• The affected side SPV and SP/AP values were both related to the degree of VH and CH.
• VD may be related to early episodic vertigo and fluctuating hearing loss in MD.
• Early detection of VD levels is crucial for patients with Meniere’s disease with EH.

## Introduction

Endolymphatic Hydrops (EH) is most prevalent in Meniere’s Disease (MD). MD is primarily characterized by fluctuating hearing loss and recurrent vertigo. Although EH is one of the most common pathological phenomena in otology, its etiology remains unclear. The development of Intravenous Gadolinium-enhanced delayed Magnetic Resonance Imaging (IV-Gd MRI) technology allows EH to be subdivided into cochlear, vestibular, and semicircular canal hydrops, and also enables quantification into different grades and scores [1]. This technique improves the relevance and reliability of EH research in clinical practice [2]. MRI shows that 44%–75% of asymptomatic ears in unilateral MD patients have EH [3], suggesting that these ears also exhibit pathological changes.

EH may be associated with various immune-related diseases, such as autoimmune arthritis, psoriasis, and irritable bowel syndrome [4]. Therefore, it is regarded as an autoimmune disorder [5]. The therapeutic effect of Vitamin D (VD) on autoimmune diseases, such as multiple sclerosis, demonstrates its powerful immune regulatory function [6]. Circulating VD and its metabolites bind to VD-binding protein (DBP), which participates in inflammatory and immune regulatory processes [7]. Chiarella G et al. reported that serum DBP levels were reduced in patients with MD [8]; meanwhile, other researchers found that VD supplementation can improve hearing in Sudden Sensorineural Hearing Loss (SSNHL) patients [9], reduce vertigo symptoms in MD patients, and decrease the frequency of intratympanic gentamicin injections [10]. A matched case-control study confirmed that serum VD levels in MD patients were significantly lower than those in individuals without MD [11]. However, a comprehensive research on the impact of VD on EH assessment remains limited.

We investigated the impact of VD on cochlear and vestibular hydrops. Additionally, we explored the pathogenesis of EH by integrating clinical imaging and electrophysiological examinations to better understand the clinical manifestations of EH.

## Methods

### Participants

**Fig. 1:**
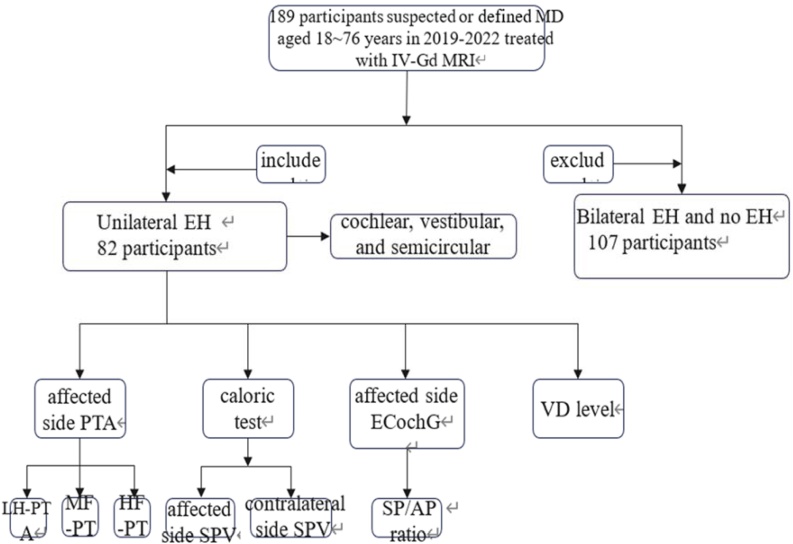
Flow chart of the included and excluded population and examination in this study. MD, Meniere’s Disease; IV-Gd MRI, Intravenous Gadolinium-enhanced delayed Magnetic Resonance Imaging; LH-PTA, Low-frequency Pure Tone Audiometry; MF-PTA, Middle-Frequency PTA; HF-PTA, High-Frequency PTA; SPV, Slow Phase Velocity; SP/AP, Summating Potential to Action Potential; VD, Vitamin D.

This cross-sectional study was approved by the Medical Ethics Committee of our institution (Clinical Trial nº SWYX: NO.2022-100), and informed consent was obtained from all participants. Overall, 189 patients diagnosed with suspected or defined MD at the vertigo clinic of our institution between 2019 and 2021 were included. Routine gadolinium-based enhanced MRI of the inner ear was performed, and unilateral EH patients were screened for inclusion. All patients were diagnosed according to the guidelines for MD diagnosis and treatment [12]. Pure Tone Audiometry (PTA), serum VD (25-Hydroxyvitamin D3 [25OHD3]) test, caloric test, and Electrocochleography (ECochG) were conducted (Fig. 1). No spontaneous nystagmus was observed during the examination. Patients with metabolic diseases that affect VD absorption and calcium metabolism were excluded. 82 ears affected by EH were included.

### Gadolinium-enhanced MRI and the reconstruction method

**Fig. 2:**
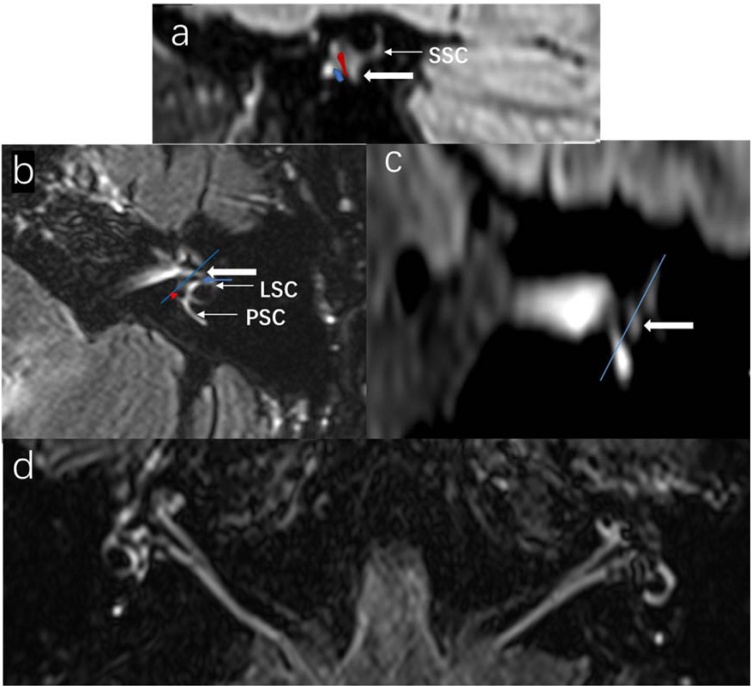
Coronal reconstruction of the SSC level showing the proportion of the utricle and saccule. The size of the saccule and the utricle is displayed in the coronal plane and LSC plane: (a) Sagittal plane reconstruction, (b) Axial plane (LSC plane), (c) Coronal plane. (d) Bilateral contrast showing endolymphatic hydrops. The right vestibule and cochlea were normal. The left cochlea showed severe hydrops, with hydrops of the saccule and utricle in the vestibular area and no enhancement of the perilymph, indicating severe endolymphatic hydrops. SCC, Superior Semicircular Canal; PSC, Posterior Semicircular Canal; LSC, Lateral Semicircular Canal; Red area, Utricle; Blue area, Saccule; Thick arrow, Vestibular area; Blue arrow, Saccule; Red triangle, Utricle.

All patients received a double dose (0.2 mmoL/kg) of Gd-DTPA dimeglumine injection (D-13342, Berlin, Germany) through the elbow vein at the standard injection rate. This was followed by intravenous administration of 20 mL normal saline solution. Imaging was performed using a 3.0-T Philips MR system equipped with a 32-channel head and neck coil. The sequence parameters for the MR examination were as follows: TR = 7600 ms, TE = 365 ms, TI = 2300 ms, and isotropic voxel size = 0.8 mm for acquisition and 0.4 mm for reconstructions. Three perpendicular semicircular canals were used to establish coordinates, with the Horizontal Semicircular Canals (HSC) serving as the X-axis and the superior semicircular canal as the Y-axis. The relationship between the saccule and utricle was confirmed using oblique sagittal plane reconstruction. The posterior semicircular canal represented the z-axis, corresponding to coronal in axial and oblique sagittal views, to minimize errors (Fig. 2a‒c) [1,13]. Signal intensities of the endolymph and perilymph were compared in both ears before and 4 h after the injection (Fig. 2d).

### Imaging grading of EH

EH degree was assessed using the visual scoring method as previously described [14,15]. The imaging level (complete, partial, or absent) of the vestibule, cochlear, and three semicircular canal hydrops was individually evaluated and incorporated. The total score ranged from 0 (no hydrops) to 18 (complete hydrops; Supplementary Table 1) [14]. VH grading was determined based on the ratio of EH area between the saccule and utricle [16]. The grading for VH and CH was as follows: 0, normal; 1, mild hydrops; 2, severe hydrops (Supplementary Materials). All imaging assessments were evaluated by two chief radiologists specializing in head and neck surgery with over a decade of experience.

**Table 1:** Clinical characteristics of the analyzed patients’ population with Meniere’s disease.

Variable	Number	Mean	SD
Sex			
Female	48		
Male	34		
Cochlear hydrops			
No	15		
Mild	45		
Severe	22		
Vestibular hydrops			
No	16		
Mild	41		
Severe	25		
Mean age, y	82	51.01	14.04
Mean PTA, dB			
LF-PTA	82	51.61	23.11
MF-PTA	82	55.38	23.34
HF-PTA	82	56.76	26.54
Caloric test, °/s			
Affected side SPV	82	13.17	10.3
Contralateral side SPV	82	21.01	13.52
Mean VD level, ng/mL	82	19.67	6.16
SP/AP ratio	44	0.36	0.19
Hydrops score	82	10.45	3.51

LH-PTA, Low-Frequency Pure Tone Audiometry; MF-PTA, Middle-Frequency PTA; HF-PTA, High-Frequency PTA; SPV, Slow Phase Velocity; SP/AP, Summating Potential to Action Potential; VD, Vitamin D.

## PTA

Patients underwent testing in a standard soundproof chamber using the Intelligent Hearing Systems PTA machine (IHS, Miami, FL, USA). The maximum detection threshold for air conduction hearing was measured at 100 dB. In cases with no response at 100 dB, the hearing threshold was adjusted to 105 dB. PTA was assessed at 125, 250, 500, 1000, 2000, 4000, and 8000 Hz. The average values of the thresholds at 125, 250, and 500 Hz were recorded as Low-Frequency PTA (LF-PTA), while those at 1000 and 2000 Hz were recorded as Middle-Frequency PTA (MF-PTA). The average auditory thresholds at 4000 and 8000 Hz were calculated for High-Frequency PTA (HF-PTA).

### Caloric test

We used the American MMT Visual Eyes nystagmus and ATMOS hot and cold stimulators to conduct the caloric test at a room temperature of < 25 °C. During the test, the subject's head was elevated to form a 30 ° angle with the horizontal plane, ensuring that the horizontal semicircular canal was perpendicular to the ground. The alternating cold and hot air irrigation method was used, with each irrigation lasting one minute. Subsequently, the irrigation sequence was Right ear hot air (RW) – Right ear Cold air (RC) – Left ear hot air (LW) – Left ear Cold air (LC), with a minimum interval of 2 minutes between each. The temperature of the cold air was 24 °C, and that of the hot air was 50 °C. Videonystagmography (VNG) was used to synchronously record and analyze the data. Generally, the absolute values of Peak Slow Phase Velocity (PSPV) on each side was summed to obtain the Slow Phase Velocity (SPV), which served as the parameter to evaluate the function of one semicircular canal. Specifically, the right-side SPV and the left-side SPV were calculated as follows: SPV(R) = PSPV(RW) + PSPV(RC); SPV(L) = PSPV(LW) + PSPV(LC). The PSPV is defined as the average velocity over a 10-second interval that includes 5-seconds before and 5-seconds after the peak nystagmus response. Additionally, this value is automatically recorded by the system and may be manually calibrated to ensure accuracy.

## VD

A fully automated electrochemiluminescence immunoassay system [17], the Cobas modular E170 (Roche, Mannheim, Germany), was used to detect VD levels with fasting venous blood collected in the morning. Serum 25OHD3 is the primary constituent of 25-hydroxyvitamin D, and its concentration accurately reflects the overall nutritional status of individuals regarding their VD levels [18]. According to laboratory data, the normal range for VD level is serum 25OHD3 ≥ 20 ng/mL; levels between 10 and 20 ng/mL are considered insufficient, and levels < 10 ng/mL indicate a deficiency [17].

### Electrocochleography

ECochG was performed using an IHS machine (IHS, Miami, FL, USA). During the test, the recording electrode was placed on the eardrum, the reference electrode was positioned on the ipsilateral earlobe, and the ground electrode was located at the center of the forehead. A short acoustic stimulus (click) with an intensity of 90 dB was presented using alternating positive and negative polarities. The compound Action Potential (AP) and Summating Potential (SP) waveform were derived by subtracting responses elicited by opposite-polarity stimuli to effectively cancel the CM. We measured the SP/AP amplitude. A value above 0.4 indicated possible hydrops.

### Statistical analysis

Data analyses were performed using SPSS 19.0 software (IBM Corp., Armonk, NY, USA). A *t*-test was conducted to examine the difference in SPV values between the two ears. The correlation between hydrops grade and each variable was assessed using the Kruskal-Wallis nonparametric test and one-way ANOVA test.

## Results

**Fig. 3:**
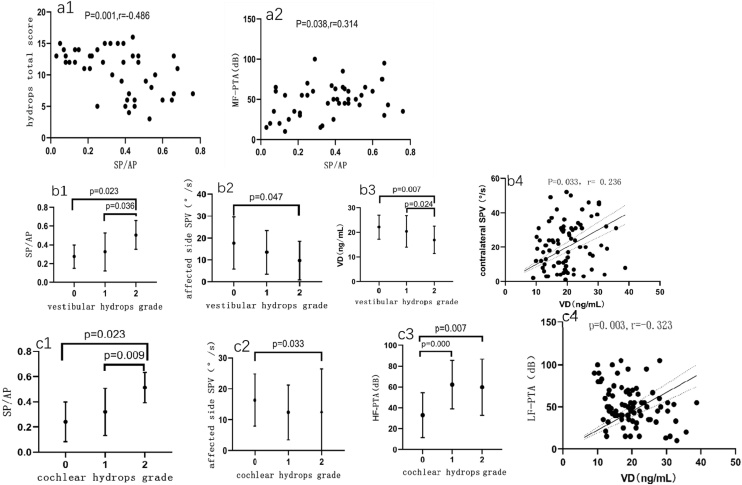
(a1) Correlation between SP/AP ratio and total hydrops score. (a2) Linear correlation between SP/AP ratio and MF-PTA. Relationship between variables and vestibular hydrops grade. (b1) Correlation between the grade of vestibular hydrops and SP/AP ratio (p = 0.013). (b2) SPV of affected ear showed a statistically significant difference among vestibular hydrops grades (p = 0.027). (b3) The mean VD level difference between the three groups was statistically significant (p = 0.016). (b4) Linear correlation between VD and contralateral ear SPV. Relationship between variables related to the cochlear hydrops grades. (c1) Correlation between cochlear hydrops grade and SP/AP ratio (p = 0.005). (c2) A statistically significant difference was observed in affected ear SPV among cochlear hydrops grades (p = 0.023). (c3) There was a statistically significant difference in HF-PTA among the three grades (p = 0.001). (c4) Linear negative correlation between VD and LF-PTA. VD, Vitamin D; SP/AP, Summating Potential/Action Potential; SPV, Slow Phase Velocity; 0, No hydrops; 1, Mild hydrops; 2, Severe hydrops; LF-PTA, Low-Frequency Pure Tone Audiometry; MF-PTA, Mid-Frequency Pure Tone Audiometry; HF-PTA, High-Frequency Pure Tone Audiometry.

We included 82 patients (34 males, 48 females) aged 15–76 years (mean age: 50.9-years). Clinical characteristics of the analyzed patients are shown in Table 1. The mean VD level was 19.67 ± 6.16 ng/mL, with the affected ear showing an SPV of 13.17 ± 10.3 °/s and the contralateral ear exhibiting an SPV of 21.01 ± 13.52 °/s. A *t*-test showed significant differences between the two ears (p = 0.016). The hydrops score ranged from 3- to 17-points (average 10.45 ± 3.51). ECochG responses were observed in 44 cases and absent in 38 cases. The mean SP/AP ratio was 0.36 ± 0.19 in 44 cases. A negative correlation was observed between SP/AP ratio and hydrops score (*r* = -0.49; Fig. 3a1). There was a linear correlation between the SP/AP ratio and MF-PTA (*r* = 0.31; Fig. 3a2). The correlations between the examinations and the level of the VH and CH were shown in Table 2.

**Table 2:** Correlation between the examinations and the grade of vestibular and cochlear hydrops.

	Cochlear hydrops			p	Vestibular hydrops			p
Grade	0	1	2		0	1	2	
Cases	15	45	22		16	41	25	
Sex (Male /Female)	3/12	22/23	9/13	0.14	8/8	16/25	10/15	0.74
Age	49.2 ± 14.11	49.64 ± 15.02	44.36 ± 11.35	0.23	53.25 ± 11.76	49.95 ± 15.57	51.6 ± 13	0.79
LF-PTA	41.93 ± 14.01	52.16 ± 24.35	57.09 ± 24.26	0.11	40.68 ± 21.55	51.29 ± 24.92	59.12 ± 18.41	0.06
MF-PTA	47.2 ± 23.91	57.2 ± 23.16	57.22 ± 23.18	0.24	45.81 ± 21.55	55.66 ± 26.49	61.04 ± 16.86	0.12
HF-PTA	33 ± 21.52	62.22 ± 23.11	61.77 ± 28.21	0.001^a^	50.63 ± 27.95	55.36 ± 29.43	62.96 ± 19.41	0.76
Affected side SPV	16.33 ± 8.47	12.33 ± 8.88	12.73 ± 23.7	0.038^a^	17.17 ± 11.90	13.49 ± 9.93	9.72 ± 8.88	0.027^a^
Contralateral side SPV	23.13 ± 12.63	19.07 ± 12.7	23.54 ± 15.57	0.419	21.81 ± 15.59	21.24 ± 12.25	20.01 ± 13.52	0.84
Mean VD level (ng/mL)	20.2 ± 6.78	20.29 ± 6	18.03 ± 6.04	0.36	22.14 ± 4.89	20.38 ± 6.46	16.91 ± 5.56	0.016^a^
VD (Normal/abnormal)	7/8	23/22	7/15	0.33	10/16	20/21	7/18	0.08
Number of SP/AP cases	9	22	13		11	21	12	
SP/AP	0.28 ± 0.21	0.31 ± 0.17	0.51 ± 0.13	0.005	0.29 ± 0.14	0.32 ± 0.21	0.49 ± 0.14	0.013^a^

LF-PTA, Low-Frequency Pure Tone Audiometry; MF-PTA, Middle-Frequency Pure Tone Audiometry; HF-PTA, High-Frequency Pure Tone Audiometry; SPV, Slow Phase Velocity; VD, Vitamin D; SP/AP, Summating Potential to Action Potential.

Normal: VD ≥ 20 ng/mL; Abnormal: VD < 20 ng/mL; ^a^ p < 0.05.

In 44 patients with elicited ECochG, VH levels were correlated with the SP/AP ratio (*U* = 8.64, p = 0.013, Kruskal-Wallis test). The SP/AP ratio of group 2 significantly differed from that of groups 0 and 1 (p = 0.005 and 0.025, respectively), with no significant difference between groups 0 and 1 (Fig. 3b1). The affected ear SPV differed significantly among the VH groups (*U* = 7.26, p = 0.027, Kruskal-Wallis test), notably between groups 2 and 0 (p = 0.047). However, no significant differences were observed between groups 0 and 1 and between groups 2 and 1 (Fig. 3b2). The average difference in VD levels was statistically significant among the three VH grading groups (*F* = 4.394, p = 0.016). Specifically, a significant difference was observed between groups 0 and 2 (p = 0.007) and between groups 0 and 1 (p = 0.024), but no difference between groups 1 and 2 (Fig. 3b3). Serum VD level and contralateral ear SPV exhibited a positive linear correlation (*r* = 0.236, p = 0.033, Spearman's rank test, Fig. 3b4).

In 44 patients with induced ECochG waveforms, the CH level and SP/AP ratio correlated significantly (*U* = 10.68, p = 0.005, Kruskal-Wallis test). Significant differences were observed between Group 2 and Group 1, and between Group 2 and Group 0 (p = 0.008, p = 0.003). However, no correlation was found between groups 0 and 1 (Fig. 3c1). The affected ear SPV differed significantly among the CH grading groups (*U* = 6.54, p = 0.038, Kruskal-Wallis test), particularly between groups 0 and 2 (p = 0.033). Differences between the other groups were not significant (Fig. 3c2). The HF-PTA differed significantly among the three CH groups (*U* = 15.06, p = 0.001, Kruskal-Wallis test), with group 0 differing significantly from Groups 1 and 2 (p = 0.000 and 0.007, respectively). However, Groups 1 and 2 did not differ significantly (Fig. 3c3, p > 0.05). Our findings revealed a significant inverse relationship between VD levels and LF-PTA (*r* = -0.323, p = 0.003, Fig. 3c4, Spearman's rank test). However, no direct association was observed between VD levels and CH grade or total CH scores.

## Discussion

In this study, the affected side SPV and SP/AP values were both related to the degree of VH and CH. The affected side SPV was correlated with the degree of hydrops in the no hydrops and severe hydrops groups in VH and CH. Unlike the affected side SPV, the difference in SP/AP value between the severe hydrops group and the other two groups was significant, indicating its higher sensitivity to changes in hydrops. The SP/AP ratio was linearly correlated with MF-PTA and total hydrops score, with correlation coefficients of 0.314 and -0.486, respectively. Thus, the SPV and SP/AP ratio on the affected side can better reflect the situation of CH and VH. Similarly, in the correlation study of hydrops, the VD level was significant only in the severe VH and no hydrops groups. Conversely, HF-PTA was significant only in the no hydrops and CH hydrops groups. These findings suggest that different stages and parts of hydrops lead to different sensory nerve affection and diverse clinical manifestations.

The inner ear plays an important role in the immune response, with various immune active cells in the endolymphatic sac and surrounding areas. VD regulates the production of immune factors by macrophages and monocytes through the VD receptor. This receptor mediates autoimmune responses [7] and enhances the bidirectional permeability of the endolymphatic vessels to induce EH [19]. and peripheral vestibular ganglion inflammatory reaction [20]. This decreases nerve conduction, which manifests as a decrease in the response of the horizontal semicircular canal and ampullary labyrinth to cold and heat stimuli. SPV during episodes and breaks can accurately reflect vertigo severity in MD patients primarily presenting with dizziness [21]. To evaluate systemic immune responses, we compared SPV values of the affected and contralateral ears and observed that the contralateral ear SPV values were positively correlated with VD in EH. The average value of the contralateral SPV was (20.12 ± 2.18), which was higher than that of the affected ear but lower than the average value for healthy individuals (34.3–35.2 ± 17.4) [22]. Our study demonstrated that the VD level exhibited a linear correlation with the contralateral SPV (*r* = 0.24), further supporting this theory. In non-hydrops ears, VD affected the integrity of the Vestibulo-Ocular Reflex (VOR) pathway neural conduction function. That could explain why 90% of the affected ear and 22% of the opposite ear in MD patients exhibited EH [23]. Therefore, prolonged duration, severe intensity, and fluctuating vertigo attacks in early VH patients may be attributed to bilateral damage to the VOR and the relatively mild initial stage of damage where function remains intact, making patients more sensitive to changes in EH. Furthermore, our analysis revealed a significant inverse correlation between the hydrops score and the SP/AP value, and when the HSC was hydrops, the utricle and saccule were mostly fused as shown by MRI. Our study clearly confirmed this hypothesis by demonstrating a clear reduction in SPV values on the affected side and VD levels during severe VH. Robert et al. discovered the fusion of the HSC and ampullary ampulla in MRI of four DEH patients. EH severity was positively correlated with higher Canal Paresis (CP) values of the caloric test, with CP asymmetry reaching up to 99% [24]. This aligns with our findings; however, their study was limited to a single case report. In contrast, our study included a larger sample size and a more comprehensive investigation.

We observed for the first time that a decrease in VD value is associated with an increased likelihood of severe VH. Given its critical role in the immune function of the inner ear, we propose that VD exerts its influence on episodic vertigo and predominantly contributes to the early progression of VH by acting on the vestibular-cochlear pathway. The absence of hydrops in any of the contralateral ears in this study suggests that attenuated VOR due to early VD deficiency did not immediately lead to EH. Raymond et al. [25] observed the emergence of novel synapses between neurons in the vestibular nucleus and intra-vestibular connections after unilateral vestibular dysfunction, a phenomenon referred to as vestibular auto-compensation. We believe that this function also plays a role in the development of hydrops, compensates for immune decompensation caused by VD deficiency, and thus can account for recurrent vertigo observed in clinical practice, specifically, the process of vestibular function compensation to establish a new equilibrium.

VD acts as an endogenous synthetic hormone, potentially due to its immunomodulatory effect on inflammation [24]. The VD level is lower in SSNHL patients than in healthy controls, and SSNHL patients with VD deficiency have worse prognosis than those without VD deficiency [26], attributing to the effect of VD on peripheral neuropathy. From another VD classical approach, excessive production of Transforming Growth Factor Beta (TGFβ) in mice is the underlying cause of cochlear ossification. Additionally, reducing TGFβ levels can lead to the restoration of hearing. The interaction between TGFβ and VD plays a role in regulating osteoblasts [27]. Moreover, 1,25(OH)2D3 possesses anti-inflammatory and immunomodulatory properties, capable of suppressing the production of proinflammatory cytokines and chemokines in macrophages [28]. Some scholars have suggested that VD deficiency is associated with hearing loss, MD [11], and otosclerosis, and these diseases may belong to a common category: autoimmune diseases. The effectiveness of hormone therapy in treating MD and SSNHL strongly supports this hypothesis. Dao-gong et al. confirmed this theory through the discovery that serum/glucocorticoid-inducible kinase-1 functions as a physiologic inhibitor of NLRP3 inflammasome activation and maintains inner ear immune homeostasis, reciprocally participating in models of MD pathogenesis [29]. Our research corroborated this perspective and further substantiated that the VD level demonstrated a linear relationship with LF-PTA (*r* = 0.32) in MD. In summary, we propose that VD deficiency may contribute to cochlear ossification, with this effect initially manifesting at the apex of the cochlea. Importantly, this process appears to be reversible. The previous conclusion that low VD is correlated with senile low-frequency hearing loss [30] and diabetic nephropathy [31], simultaneously, our study confirms it related to early MD. Clinically, high-frequency hearing loss in MD typically signifies that the condition has advanced to the middle or late stages, with cochlear damage progressing from the apex toward the base. Our study demonstrated a direct correlation between HF-PTA (56.76 ± 26.54) of the affected ear and CH. Attye et al. also found that saccular hydrops is associated with sensorineural hearing loss at levels above 40 dB [13]. Therefore, early fluctuating hearing loss is associated with the auditory regulation function of VD, but not with CH. This finding confirms the immunomodulatory role of VD in MD.

VD also suppresses neuroinflammation to counteract progressive spiral ganglion degeneration [32]. Therefore, we believe that a decrease in VD levels weakens those effects, resulting in heightened vascular permeability, damage to the ultrastructure of the labyrinthine barrier within the inner ear membrane, and exacerbation of hydrops.

Therefore, early detection of VD levels is important for promoting hearing recovery, and VD supplementation can effectively rectify neuroepithelial degeneration in the inner ear of EH patients, thereby slowing disease progression.

Our selection criteria were directly based on the MR imaging manifestation of MD. The selected patients were in the stable disease stage to minimize potential interference variables as much as possible. However, the ECochG extraction rate was low (53.6%), and the caloric test CP value could not be measured when the bilateral SPV value was less than 12 °/s. Therefore, we further refined the caloric test results into the SPV values of the affected and contralateral ears, which can accurately identify patients with potential vestibular organ lesions causing vertigo [22].

Although this study adheres to strict admission and testing standards, it has certain limitations. Owing to ethical constraints, administering enhanced gadolinium to a group of normal healthy volunteers in order to observe the characteristics of EH is not permitted. Therefore, healthy controls with the same VD level were not established. The effect of VD varies based on the severity and duration of deficiency. Future studies should focus on identifying individuals with initial-stage VD deficiency in mild hydrops and conducting follow-up examinations post-VD supplementation to support this hypothesis. Therefore, early detection of VD levels is crucial for MD patients with EH.

## Conclusions

The study found that in MD patients with unilateral EH, the severity of vestibulocochlear hydrops correlates closely with electrophysiological test results. Specifically, SPV and SP/AP ratio on the affected side are linked to vestibulocochlear hydrops, while VD is associated with contralateral SPV, VH, and LF-PTA. VD may be related to early episodic vertigo and fluctuating hearing loss in MD.

## Funding

This study was supported by the Incubation Fundation of Shandong Provincial Hospital.

## Data availability statement

The authors declare that all data are available in repository.

## Conflicts of interest

The authors declare no conflicts of interest regarding the recommendation for assessing VD levels in patients with EH.
