# Supplementary material for: Endolymphatic hydrops and its association with Magnetic Resonance Imaging and serum vitamin D levels
Source: Braz J Otorhinolaryngol. 2026 Jun 16;92(5):101858. doi: 10.1016/j.bjorl.2026.101858 (PMC13285279; doi:10.1016/j.bjorl.2026.101858)

BJORL-D-25-00078_Supplementary Material

**Supplementary Table**

**Table 1** MR Gadolinium visual scoring of endolymphatic hydrops in the inner ear.

| **Imaging** | **Cochlea** | | | **Vestibular** | **Semicircular canals** | | |
| --- | --- | --- | --- | --- | --- | --- | --- |
|  | **Basal turn** | **Middle turn** | **Apical turn** |  | **SSC** | **HSC** | **PSC** |
| Undeveloped | 0 | 0 | 0 | 0 | 0 | 0 | 0 |
| Partially developed | 2 | 1 | - | 3 | 1 | 1 | 1 |
| Fully developed | 3 | 2 | 1 | 6 | 2 | 2 | 2 |

SSC, Superior Semicircular Canal; HSC, Horizontal Semicircular Canal; PSC, Posterior Semicircular Duct.

**Supplementary Materials**

**Figure A** Vestibular hydrops grade 0. (a) The saccule is smaller than the utricle in the horizontal semicircular canal plane; (b) The size of the saccule and the utricle is shown in the coronal plane. Thin arrow: cochlea; Thick arrow: vestibular area; Red area: utricle; Blue area: saccule.


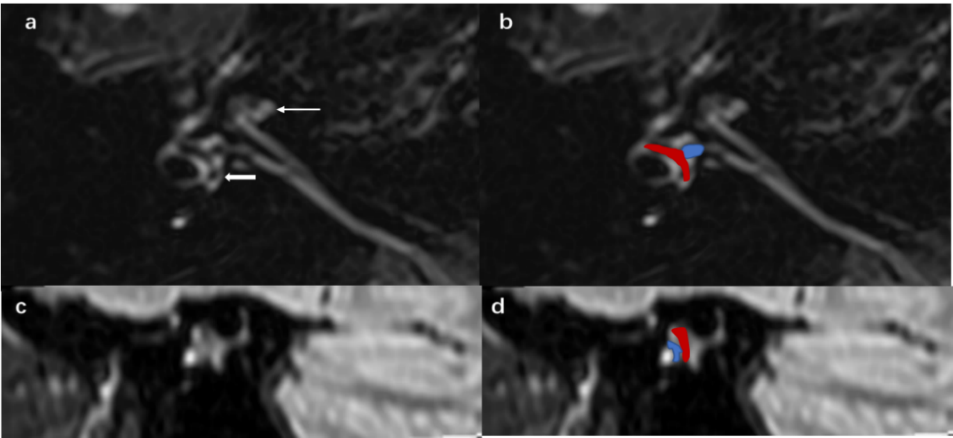


**Figure B** Vestibular hydrops grade 1 (a–b) The saccule increased to almost the size of the utricle, SURI=1; (c–d) The saccule is larger than the utricle but not fused, and the perilymph is clear and visible, SURI >1; (e) The sagittal plane shows hydrops in the saccule and utricle. Thick arrow: vestibular area; Red area: utricle; Blue area: saccule.


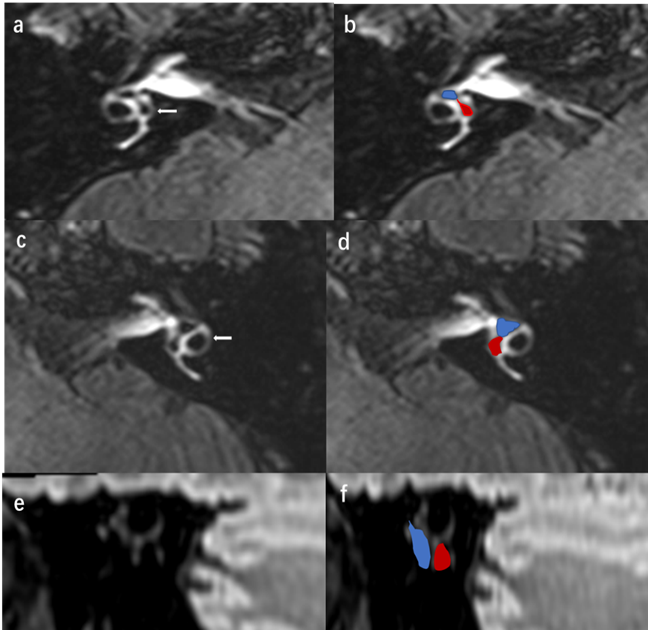


**Figure C** Vestibular hydrops grade 2. The utricle and saccule were fused, and the saccule could not be identified. (a‒b) Compression of the perilymph in the vestibular area; (c‒d) Enhanced visualization of the horizontal semicircular canals. Blue area: vestibular endolymph; Thick arrow: vestibular area; LSC, Lateral Semicircular Canal.


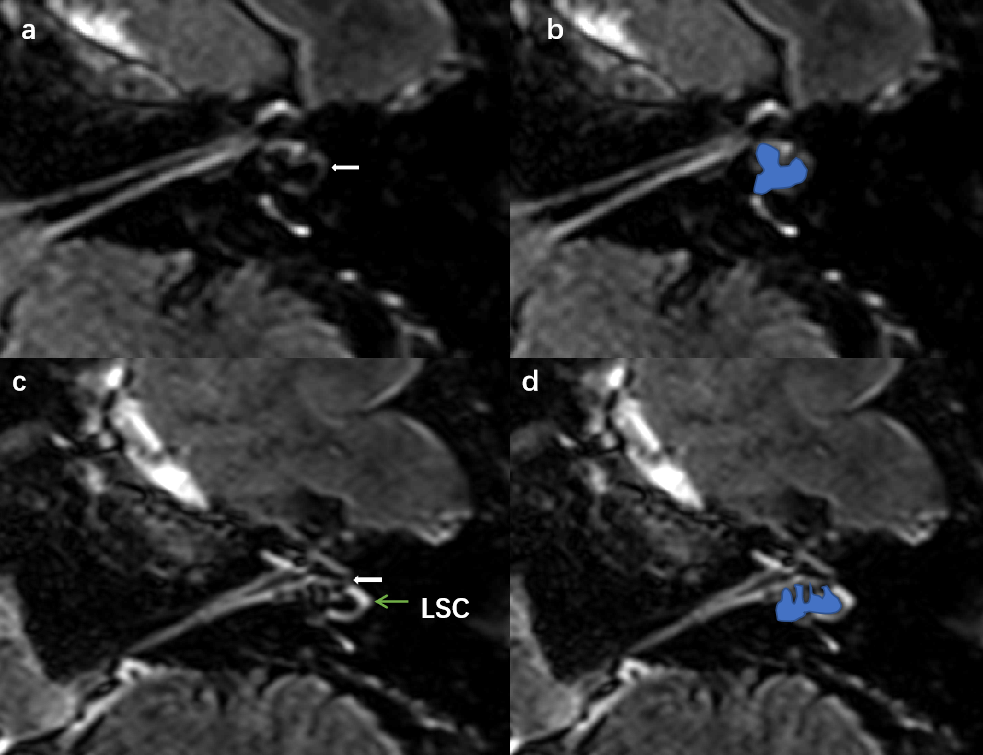


**Figure D** Cochlear hydrops (a) Normal cochlear with no hydrops (grade 0); (b–c) Cochlear hydrops grade 1, yellow marks represent scala vestibuli (arrow), which is fully obliterated. (d–e) Cochlear hydrops grade 2, yellow marks represent scala tympani hydrops. The scala vestibuli and the bottom turn are fully obliterated, the intermediate turn is partially obliterated, and the top turn is not obliterated.


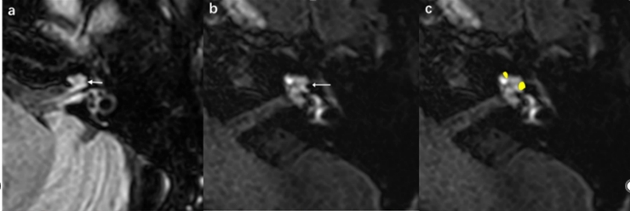

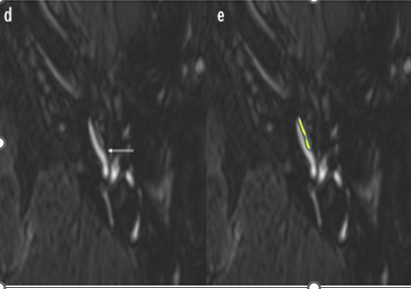

Supplement: Supplementary file 1 [file mmc1.docx]
